# The Interaction of Craniofacial Morphology and Body Mass Index in Obstructive Sleep Apnea

**DOI:** 10.3390/dj10070136

**Published:** 2022-07-19

**Authors:** Federica Bertuzzi, Antonio Santagostini, Matteo Pollis, Fabio Meola, Marzia Segù

**Affiliations:** 1 Department of Clinical-Surgical, Diagnostic and Pediatric Sciences, Section of Dentistry of Pavia, University of Pavia, 27100 Pavia, Italy; federica.bertuzzi01@universitadipavia.it; 2 Department of Medicine and Surgery, University of Parma, 43126 Parma, Italy; marzia.segu@unipr.it; 3 School of Dentistry, University of Siena, 53100 Siena, Italy; matteo.pollis@gmail.com; 4 Spino D’Adda, 26016 Cremona, Italy; info@outsideformat.com

**Keywords:** obstructive sleep apnea, cephalometric analysis, polysomnography

## Abstract

Aim: This study sets out to explore the relationship between craniofacial morphology and obstructive sleep apnea (OSA) severity, assessing the relative contribution of obesity, calculated using BMI. Methods: A sample of 30 adult patients (20 males; 10 females), mean age = 54(±76) years, with a polysomnography-confirmed diagnosis of OSA, i.e., with an apnea-hypopnea index (AHI) of over 5 events/h, was recruited and underwent cephalometric evaluation. Sleep parameters, namely AHI, AHI supine, oxygen desaturation index (ODI), and mean oxygen saturation [Mean SaO2%], were assessed. Correlation analysis between 13 cephalometric features and AHI was performed using a Pearson test. The sample was split into three groups based on AHI score (mild = 10 < AHI < 15; moderate = 15 < AHI < 30; severe = AHI > 30), and ANOVA was performed to compare the means of cephalometric features. In addition, the sample was split into two groups according to BMI (normal weight = BMI < 25; overweight = BMI > 25). Correlation analysis between cephalometric features and AHI was performed for each group using a Pearson test. Results: The average polysomnographic values were AHI = 29.08(±16); AHI supine = 43.45(±21); ODI = 23.98(±21); mean SaO2(%) = 93.12(±2). Posterior facial height (PFH) was significantly lower in the severe OSA group than in patients with moderate OSA (*p* = 0.05). In the normal-weight group, negative correlations of the PFH and SNA angle with AHI (r = −0.36; r = −0.25, respectively), and positive correlations of the FMA angle and MP-H distance with AHI (r = 0.29; r = 0.20, respectively), were found. In the overweight group, negative correlations of AO-BO distance, SPAS (upper posterior airway space) and PAS (posterior airway space) with AHI (r = −0.30; r = −0.28; r = −0.24, respectively), and positive correlations of AFH (anterior facial height) and the FMA angle with AHI (r = 0.32; r = 0.25, respectively), emerged. Conclusions: PFH seems to be related to the aggravation of OSA. In normal-weight subjects, hard tissue-related factors have a greater impact on OSA severity, whereas in overweight subjects, the impact of fat tissue is greater.

## 1. Introduction

Obstructive sleep apnea (OSA) is a common respiratory disorder in which repeated complete (i.e., apnea) or partial (i.e., hypopnea) upper airway obstruction during sleep is associated with phasic drops in blood oxygenation and arterial hemoglobin desaturation.

According to the literature, globally, OSA may affect around 1 billion people aged 30–69 years, and the number of people with moderate to severe OSA is estimated at almost 425 million [1]. Despite the high prevalence of the condition within the adult population, many patients remain undiagnosed.

OSA is considered an independent risk factor for clinical conditions, including systemic hypertension, cardiovascular disease, stroke, and abnormal glucose metabolism [2]. In addition, repeated obstruction and awakening events during sleep can result in fragmented sleep architecture, reducing the restorative capacity of sleep [2].

Pharyngeal airway collapse during sleep depends on a combination of anatomical and neuromuscular factors [3], and in the absence of muscle compensation may be facilitated by anatomical conditions.

Epidemiological studies consistently identified obesity as the strongest risk factor for OSA [2] while factors, such as craniofacial anomalies, age, sex, and tonsillar and adenoid hypertrophy, may have a crucial predisposing effect. Numerous radiographic imaging studies reported an increased tendency of OSA subjects to display narrow and elongated airways, micrognathia, retrognathia, soft palate elongation, macroglossia, and a low-set hyoid bone [3,4,5,6,7]. In addition, many studies reported a relationship between craniofacial anomalies and OSA, especially in non-obese subjects [8].

Latero-lateral teleradiography of the head has been adopted by many authors to evaluate craniofacial anatomy. Teleradiography is a reliable and simple examination that delivers a lower radiation dose than other methods used for the evaluation of craniofacial anatomy (e.g., computerized tomography) [4]. It allows the evaluation of facial anatomy through cephalometric analysis of hard tissues. Soft tissues play a significant role in the onset of OSA events, and teleradiography is sufficiently reliable in soft tissue assessment to allow identification of sites of obstruction on a sagittal plane. For these reasons, in some centers, cephalometric analysis together with endoscopic evaluation is part of the standard assessment of airway anatomy [4].

In this study, the anatomical risk factors for OSA were investigated and their contribution to the severity of the disease was assessed. The aim of the study is to explore the relationship between craniofacial morphology and OSA severity, assessing the relative contribution of obesity.

## 2. Materials and Methods

The study was approved by the Unit internal review board (17–1023). All the participating patients were selected by a dentist with expertise in sleep medicine, on the basis of medical, psychological, and dental criteria. None had previously received treatment for OSA. Thus, a sample of 30 adult patients, 20 males and 10 females (mean age = 54 ± 7.6 years), each with a polysomnography (PSG)-confirmed diagnosis of OSA, was recruited and underwent latero-lateral teleradiography of the head followed by two cephalometric analyses: Tweed’s analysis and analysis of upper airway morphology.

Exclusion criteria were a history of upper airway or maxillofacial surgery, craniofacial syndromes, head–neck neoplasia, a number of apnea–hypopnea events per hour (apnea–hypopnea index [AHI]) lower than 5 events/h, and age < 18 years. Sleep parameters, namely AHI, AHI supine, oxygen desaturation index (ODI), and mean SaO2(%), were assessed. Body mass index (BMI) was calculated in the individual subjects.

All patients underwent single-night PSG recordings in a sleep laboratory setting. OSA severity was evaluated by measuring the number of apnea and hypopnea events per hour. In accordance with the criteria established by the American Academy of Sleep Medicine [1], a >90% drop in flow amplitude compared with the pre-event baseline for at least 10 s was considered an apnea, while a ≥50% drop in flow amplitude with respect to the pre-event baseline for at least 10 s associated with an at least 3% reduction in saturation was taken as an episode of hypopnea.

Latero-lateral teleradiography was performed in all the patients by experienced radiology technicians. DeltaDent CE software was used by a certified orthodontist (D.M.) to assess 13 cephalometric variables.

In particular, Tweed’s analysis was performed to evaluate (Table 1):

- the SNA angle, SNB angle, ANB angle: sagittal facial projection
- the FMA angle: between the mandibular plane (Go-Me) and the Frankfurt plane
- the occlusal plane angle: formed between the Frankfurt plane and the occlusal plane (OCLP-OCLA)
- AO-BO: the millimeter distance in the occlusal plane between the orthogonal projections of points A and B.
- PFH (posterior facial height)
- AFH (anterior facial height).

Analysis of upper airway morphology was performed to evaluate:

- SPAS: the thickness of the airway behind the soft palate along a line parallel to the Go-point B plane.
- PAS: (oropharyngeal space): linear distance between a point at the base of the tongue and another point on the posterior wall of the pharynx, both measured by the extension of a line from point B to point Go.
- MP-H: linear distance between H, the most anterosuperior point of the hyoid bone, and the mandibular plane measured perpendicularly to the latter.
- PNS-P: soft palate length.
- C3-H: linear distance between points C and H, where C3 is the most anteroinferior point of the third cervical vertebra.

Statistical analysis for each cephalometric variable, the mean value (+/− standard deviation) in the study sample was calculated. Correlation analysis between cephalometric features and AHI was performed by means of a Pearson test. The sample was split into three groups based on AHI score (mild = 10 < AHI < 15; moderate = 15 < AHI < 30; severe = AHI > 30) and ANOVA and LSD post-hoc analysis were performed.

In addition, the sample was split into two groups according to BMI (normal weight = BMI < 25; overweight = BMI > 25). Again, correlation analysis between cephalometric features and AHI was performed for each group by means of a Pearson test.

The cut-off for statistical significance was set at *p* < 0.05. All statistical procedures were performed using SPSS 27.0 software (IBM, Milan, Italy).

## 3. Results

The sample was made up of 30 subjects (20 males and 10 females) with a mean age of 54(±76) years. Based on the AHI values, 8 patients had mild OSA (AHI > 5 < 15), 9 patients had moderate OSA (AHI > 15 < 30), and 13 patients had severe OSA (AHI > 30).

The following average polysomnographic values were found in the sample: AHI = 29.08(±16); AHI supine = 43.45(±21); ODI = 23.98(±21); mean SaO2(%) = 93.12(±2).

The mean cephalometric values were: SNA angle = 81.14(±3); SNB angle = 78.27(±3); ANB angle = 2.85(±2); FMA angle = 24.01(±6); AO-BO = −0.81(±4); occlusal plane angle = 10.22(±4); PFH = 51.00(±11); AFH = 66.33(±9); SPAS = 9.94(±2); PAS = 8.56(±2); PNS-P = 30.69(±6); MP-H = 21.08(±5); H-C3 = 35.59(±6).

From the Pearson test results, none of the cephalometric variables analyzed showed a statistically significant correlation with the polysomnographic parameters considered (Table 2).

The sample was split into three groups based on AHI score (mild = 10 < AHI < 15; moderate = 15 < AHI < 30; severe = AHI > 30). ANOVA showed that PFH was eligible for post-hoc analysis (*p* = 0.138). LSD post-hoc analysis reported a significantly lower PFH in the severe OSA group than in moderate OSA patients (*p* = 0.05) (Table 3 and Table 4).

The sample was divided into two groups based on the BMI of the single patients (normal weight = BMI < 25; overweight = BMI > 25).

### 3.1. Normal-Weight Group

The normal-weight sample comprised 15 subjects (4 females, 11 males), mean age = 52.8(±9) years, with a BMI < 25 (mean BMI = 23.25(±1)). They had a mean AHI of 28.24(±18 events/h), and a mean AHI supine of 35.6(±14); their mean ODI was 20.36(±18) and mean SaO2(%) was 93.55(±2).

The mean cephalometric values were: SNA angle = 81.04(±2), SNB angle = 78.82(±3), ANB angle = 2.19(±2), FMA angle = 23.5(±5), AO-BO = −1.6(±3), occlusal plane angle = 10.18(±3), PFH = 51.52(±7), AFH = 64.72(±6), SPAS = 9.80(±2), PAS = 7.62(±1), MP-H = 20.54(±5), Pns-P = 32.24(± 7), H-C3 = 35.5(± 5).

**Figure 1:**
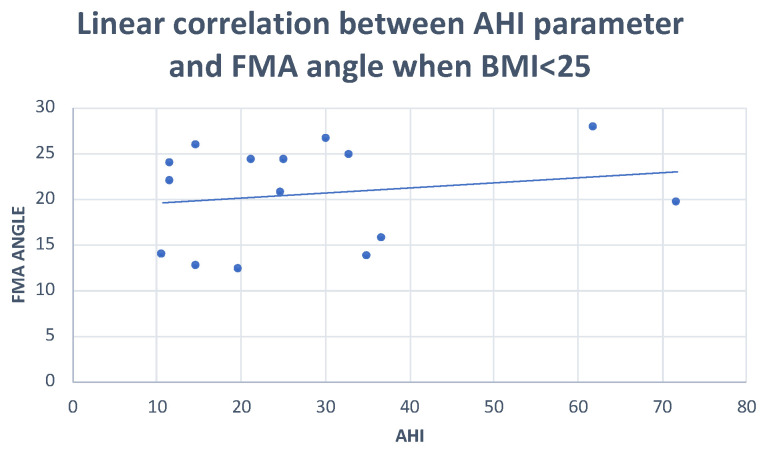
Scatter plot defining the relationship between AHI and FMA angle. r = 0.29.

**Figure 2:**
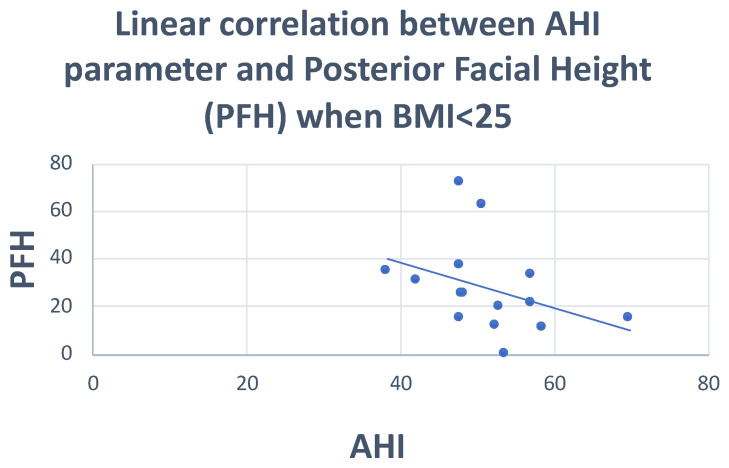
Scatter plot defining the relationship between AHI and posterior facial height. r = −0.36.

**Figure 3:**
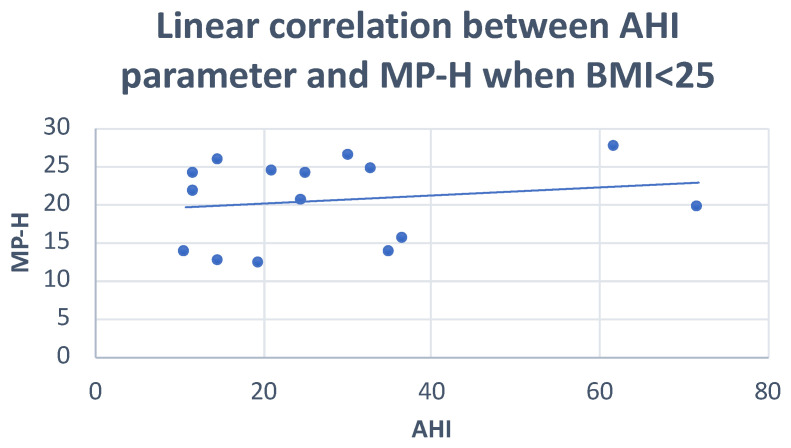
Scatter plot defining the relationship between AHI and MP-H distance. r = 0.29.

Pearson’s correlation test showed: negative correlations of the PFH and SNA angle with AHI (r = −0.36; r = −0.29, respectively), and positive correlations of the FMA angle and MP-H with AHI (r = 0.29; r = 0.20, respectively) (Figure 1, Figure 2 and Figure 3) (Table 5).

### 3.2. Overweight Group

This sample comprised 15 patients (9 males, 6 females), mean age = 56.74(±9) years, with a BMI > 25 (mean BMI = 30.26(±2)). The average polysomnographic values were: AHI = 36.3(±2), AHI supine = 51.3(±24), ODI = 28.16 (±24), Mean SaO_2_% = 92.63(±2).

The average cephalometric values were: SNA angle = 81.23(±3); SNB angle = 77.72(±3); ANB angle = 3.52(±3); FMA angle = 24.51(±7); AO-BO = 0.06(±5); occlusal plane angle = 10.26(±6); PFH = 50.48(±14); AFH = 67.95(±11); SPAS = 10.08(±2); PAS = 9.5(±2); MP-H = 21.26(±5); Pns-P = 29.14(±3); H-C3 = 36.19(±6).

**Figure 4:**
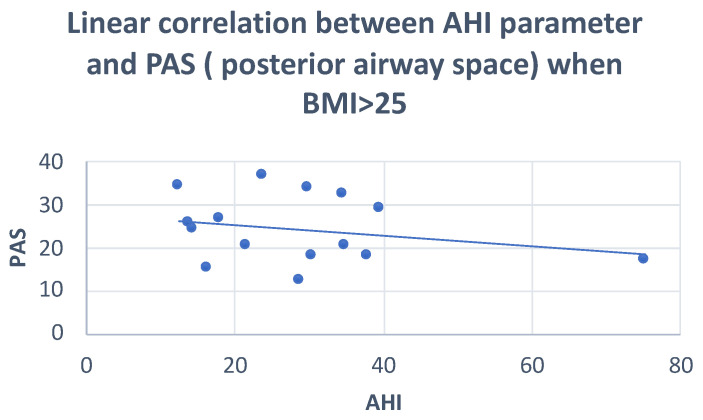
Scatter plot defining the correlation between AHI and PAS. r = −0.24.

**Figure 5:**
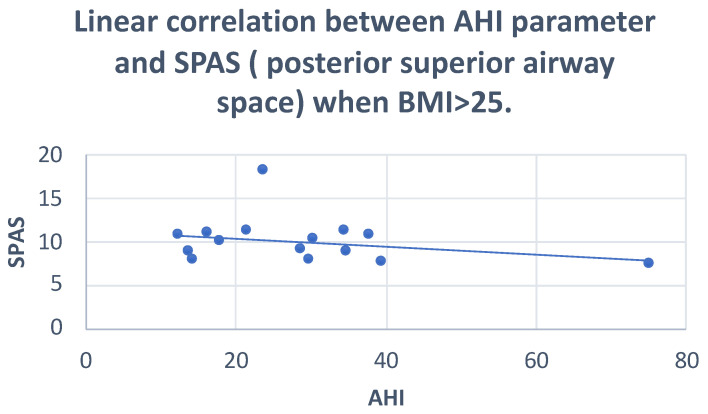
Scatter plot defining the correlation between AHI and SPAS. r = −0.28.

The Pearson correlation showed: negative correlations of AO-BO, SPAS and PAS with AHI (r = −0.30; r = −0.28; r = −0.24, respectively) and positive correlations of AFH and FMA with AHI (r = 0.32; r = 0.25, respectively) (Figure 4 and Figure 5) (Table 6).

## 4. Discussion

Obstructive sleep apnea is a relatively common sleep disorder characterized by recurrent episodes of partial or complete upper airway collapse during sleep, associated with phasic drops in blood oxygenation and arterial hemoglobin desaturation [9]. Although continuous positive airway pressure (CPAP) therapy remains the standard treatment for OSA, mandibular advancement device (MAD) treatment is increasingly seen as a valid alternative, showing greater patient compliance and remarkable efficacy in mild to moderate cases [10,11]. Use of a trial device might be a means of identifying responders to oral appliance therapy [12]. OSA may have harmful health consequences, such as cardiovascular and metabolic disorders [13]. The pathophysiology of OSA is complex with many different risk factors, both anatomical and non-anatomical, that may predispose to obstructive events. Obesity and craniofacial anomalies are considered the main anatomical predisposing factors [14,15]. In different ways, both may reduce the pharyngeal airway space, specifically through the deposition of fat tissue in obese subjects, and as an effect of hard and soft tissue-related features [4,6,16,17,18,19]. This study set out to explore, through cephalometric tracing performed on lateral projection teleradiography of the head, the relationship between craniofacial and upper airway morphology and severity of OSA, taking into account the relative contribution of obesity as a risk factor for OSA.

The results of our study showed no significant correlation between AHI and craniofacial morphology, i.e., the cephalometric variables analyzed (*p*-value > 0.05). On this basis, it seems that OSA severity is not related to a characteristic craniofacial phenotype.

However, when the sample was divided by severity of OSA, post-hoc ANOVA showed a significantly lower PFH in the severe OSA group compared with the moderate OSA patients (*p*-value = 0.05).

In addition, on splitting the sample into two groups based on BMI, in the normal-weight group (BMI < 25), AHI was found to be negatively correlated with PFH and SNA angle (r = −0.36; r = −0.25, respectively) and positively correlated with the FMA angle and MP-H (r = 0.29; r = 0.20, respectively). In the overweight group, AHI correlated negatively with AO-BO, SPAS and PAS (r = −0.30; r = −0.28; r = −0.24, respectively), and positively with the AFH and FMA angle (r = 0.32; r = 0.25, respectively).

Our findings seem to show that the worsening of OSA is related to a reduction in PFH. Analysis of the FMA angle in the patients examined suggest a tendency towards hyper-divergence, which is characterized by a progressive reduction in PFH and a corresponding increase in AFH.

Similarly, considerations made by Neelapu et al. in a literature review support the idea that the hyperdivergent facial pattern is characterized by a sagittal and vertical discrepancy, and by clockwise rotation of mandibular growth, features are in turn associated with a possible narrowing of the oropharyngeal space and an increase in anterior height of the lower third of the face [6].

A more caudal position of the hyoid bone is perhaps the parameter most recognized by authors [5,6,7].

The caudal transition of this element could determine a volume reduction of the pharynx, in turn leading to worsening OSA severity.

Our statistical analysis also detected, in the normal-weight group, a positive correlation between the AHI and the position of the hyoid bone, evaluated as the MP-H distance, (“r” = 0.29)

It can therefore be argued that as the AHI increases, so does the perpendicular distance between the hyoid bone and the mandibular plane (Go-Me), leading to a lower position of the hyoid bone in OSA subjects. Gungor et al. linked the importance of the hyoid bone to the position of the tongue: since this bone anchors the tongue muscles, its downward displacement would cause a migration of the lingual mass in the hypopharyngeal area, and therefore a reduction of the same [7]. On the other hand, other authors interpret the inferior location of the hyoid as a physiological adaptation serving to maintain the patency of the airways [4].

The role of the soft tissues as a risk factor for OSA is known. A review by Gottlieb and Punjabi supported the view that fat tissue in the lateral pharyngeal wall, soft palate length, and tongue volume play a strong role as OSA predisposing factors [20]. Increased length and thickness of the soft palate leads to a reduction of the posterior superior airway space, while increased volume of the tongue is associated with its retroposition; and therefore, with a reduction in the PAS. In our study, no significant correlation was found with the length of the soft palate.

Studies such as those by Stipa et al. and Borges et al. demonstrated that soft palate length is significantly greater in subjects with severe OSA than in subjects with mild and moderate OSA [21,22].

Other authors, such as Silva et al. [23], found that although the Psn-P value was increased in subjects with moderate and severe apnea compared with individuals with mild OSA or primary snorers, in which the increase was not statistically significant. In our study, cephalometric variables relating to the shape and volume of the tongue were not analyzed.

Instead, the correlations found in our overweight group seem to show that reductions in PAS and SPAS have a greater impact on the severity of the disease.

The results of our study suggest that an increase in BMI may be related to a reduction in posterior and nasopharyngeal airway space.

In accordance with Neelapu et al., a significant decrease in nasopharyngeal and oropharyngeal space, which could depend on the encroachment and position of other structures [6], was observed.

A tendency to the third class, in the form of a reduction in AO-BO (r = −0.30) was also noted.

On the basis of our results, we therefore suggest that craniofacial morphology is not a strong indicator for assessing the severity of OSA. However, a lower PFH may play a role in worsening OSA. Moreover, as regards the impact of BMI (an anthropometric value), in subjects with a normal BMI, disease severity appears to be related more to craniofacial skeletal anomalies. However, in subjects with a high BMI, parapharyngeal fat tissue seems to contribute to pharyngeal space narrowing, and therefore to the worsening of OSA.

In support of this, a study investigating differences in craniofacial structure between obese and non-obese OSA patients revealed a predominance of skeletal abnormalities in affected non-obese patients, while major changes in soft tissue characterized the obese patients [8].

Although there is a considerable amount of literature on the correlation between OSA and craniofacial anatomy, the results are often conflicting. The large number of types of cephalometric analysis may contribute to some of the differences between studies [24]. Dis-homogeneity of the findings may also depend on the complexity of OSA pathophysiology. Indeed, alongside the purely anatomical etiological factors analyzed in the present study, there are also purely neuromuscular ones, which could have an important role in determining aggravation of this disease.

As regards the limitations of this study, it must be pointed out that anatomical imbalance predisposing to upper airway collapse can be driven by many other different factors (e.g., ethnicity, age, and sex) which were not matched in this study. Additionally, we did not perform a priori sample size calculation, and the lack of strong significance in the results may be influenced by the small sample and low power of the study. According to the method, cephalometric analysis evaluates only the sagittal dimension, without assessing any relationship with the frontal plane. Finally, since teleradiography is a two-dimensional examination used to represent three-dimensional structures, the borders of some structures may not be sufficiently defined. Moreover, no information on pharyngeal dynamics can be obtained by teleradiography.

## 5. Conclusions

Craniofacial morphology does not appear to be a significant indicator for assessing the severity of obstructive sleep apnea (*p* > 0.05). However, posterior facial height seems to be related to aggravation of OSA. Moreover, in subjects with a normal BMI, the severity of the disease seems to be related more to craniofacial skeletal anomalies, whereas in subjects with a high BMI, parapharyngeal fat tissue seems to contribute to the narrowing of the pharyngeal space, and therefore to the worsening of OSA.

## Figures and Tables

**Table 1:** Cephalometric points considered in the study.

Sella	S	Midpoint of the sella turcica
Nasion	N	Most anterior point of the frontonasal suture
Point A	A	Deepest anterior point on the maxilla anterior concavity
Point B	B	Deepest anterior point on the mandibular symphysis
Porion	Po	Most superior point on the external auditory meatus
Pogonion	Pg	Most anterior point on the mandibular symphysis
Pterion	Pt	Most posterior superior point on the pterygomaxillary fissure
Orbitale	Or	Most inferior point on the lower border of the bony orbit
Basion	Ba	Most anterior-inferior point on the foramen magnum
Articulare	Ar	Most posterior point on the condylar neck
Gonion	Go	Point of intersection between the mandibular plane and the tangent line to the posterior mandibular border
Menton	Me	Most inferior point on the mandibular symphysis
Anterior nasal spine	ANS	Most anterior point of the hard palate
Posterior nasal spine	PNS	Most posterior point of the hard palate
Condilo	Co	Most superior point on the condylar head
Sigmoid incision	Sg	Deepest point on the sigmoid incision
Anterior occlusal point	OCLA	Midpoint of the segment joining the upper incisal point to the lower one
Posterior occlusal point	OCLP	Midpoint of the occlusal surface of the first permanent molars
Hyoid	H	Most anterior-superior point on the hyoid bone
Upper posterior airway space	SPAS	Thickness of the airway behind the soft palate along a line parallel to the Go-point B plane
Posterior airway space	PAS:	Linear distance between a point at the base of the tongue and another point on the posterior wall of the pharynx, both measured by the extension of a line from point B to point Go
Mandibular plane	MP	Plane tangent to the lower edge of the mandible passing through Go and Me
Cervical vertebra	C3	Most anteroinferior point of the third cervical vertebra
Uvula apex	P	Inferior tip of the uvula

**Table 2:** Pearson correlation between cephalometric variables and OSA parameters (AHI, AHI supine, Mean SaO2%, ODI).

		AHI	AHI Supine	Mean SaO2%	ODI
SNA angle	Pearson correlation	−0.004	−0.342	0.044	−0.068
	Sig. (2-tailed)	0.982	0.120	0.826	0.729
	N	30	22	28	28
SNB angle	Pearson correlation	0.008	−0.160	−0.103	0.038
	Sig. (2-tailed)	0.968	0.476	0.602	0.848
	N	30	22	28	28
ANB angle	Pearson correlation	−0.022	−0.171	0.180	−0.120
	Sig. (2-tailed)	0.907	0.447	0.359	0.543
	N	30	22	28	28
FMA angle	Pearson correlation	−0.018	0.043	0.161	−0.175
	Sig. (2-tailed)	0.925	0.849	0.412	0.372
	N	30	22	28	28
AO-BO	Pearson correlation	−0.080	−0.155	0.177	−0.166
	Sig. (2-tailed)	0.675	0.491	0.367	0.399
	N	30	22	28	28
Occlusal plane angle	Pearson correlation	0.032	0.091	0.076	0.003
	Sig. (2-tailed)	0.867	0.686	0.699	0.988
	N	30	22	28	28
PFH	Pearson correlation	−0.110	−0.096	−0.099	0.103
	Sig. (2-tailed)	0.564	0.671	0.616	0.601
	N	30	22	28	28
AFH	Pearson correlation	0.228	0.191	−0.235	0.397
	Sig. (2-tailed)	0.225	0.394	0.229	0.036
	N	30	22	28	28
SPAS	Pearson correlation	−0.148	0.142	0.092	−0.159
	Sig. (2-tailed)	0.436	0.529	0.640	0.420
	N	30	22	28	28
PAS	Pearson correlation	−0.068	0.064	−0.070	0.013
	Sig. (2-tailed)	0.720	0.779	0.725	0.947
	N	30	22	28	28
Pns-P	Pearson correlation	0.147	−0.183	−0.080	0.082
	Sig. (2-tailed)	0.439	0.414	0.687	0.679
	N	30	22	28	28
MP-H	Pearson correlation	0.013	0.287	−0.136	0.053
	Sig. (2-tailed)	0.945	0.196	0.489	0.788
	N	30	22	28	28
H-C3	Pearson correlation	0.106	0.087	−0.216	0.213
	Sig. (2-tailed)	0.578	0.700	0.269	0.276
	N	30	22	28	28

**Table 3:** ANOVA of cephalometric variables in three OSA severity groups (1 = mild OSA; 2 = moderate OSA; 3 = severe OSA).

ANOVA						
		Sum of Squares	df	Mean Square	F	Sig.
SNA angle	Between groups	17,051	2	8525	0.938	0.404
	Within groups	245,381	27	9088		
	Total	262,432	29			
SNB angle	Between groups	11,414	2	5707	0.433	0.653
	Within groups	355,725	27	13,175		
	Total	367,139	29			
ANB angle	Between groups	853	2	426	0.052	0.949
	Within groups	219,421	27	8127		
	Total	220,274	29			
FMA angle	Between groups	4312	2	2156	0.045	0.956
	Within groups	1,302,595	27	48,244		
	Total	1,306,907	29			
AO-BO	Between groups	7748	2	3874	0.175	0.840
	Within groups	597,539	27	22,131		
	Total	605,287	29			
Occlusal plane angle	Between groups	2350	2	1175	0.046	0.955
	Within groups	682,843	27	25,290		
	Total	685,194	29			
PFH	Between groups	506,211	2	253,105	2.132	0.138
	Within groups	3,205,448	27	118,720		
	Total	3,711,659	29			
AFH	Between groups	111,739	2	55,870	0.632	0.539
	Within groups	2,387,451	27	88,424		
	Total	2,499,190	29			
SPAS	Between groups	6855	2	3427	0.547	0.585
	Within groups	169,140	27	6264		
	Total	175,995	29			
PAS	Between groups	11,703	2	5852	1.225	0.310
	Within groups	129,006	27	4778		
	Total	140,710	29			
MP-H	Between groups	5243	2	2622	0.079	0.925
	Within groups	900,285	27	33,344		
	Total	905,528	29			
Pns-P	Between groups	40,620	2	20,310	0.540	0.589
	Within groups	1,015,350	27	37,606		
	Total	1,055,970	29			
H-C3	Between groups	15,113	2	7557	0.198	0.821
	Within groups	1,029,136	27	38,116		
	Total	1,044,250	29			

**Table 4:** LSD post-hoc analysis between posterior facial height (PFH) and OSA severity.

Dependent Variable	(I) AHI_NOM	(J) AHI_NOM	Mean Difference (I-J)	Std. Error	Sig.	95% Confidence Interval	
						Lower Bound	Upper Bound
PFH	1	2	−4.6806	5.2944	0.384	−15.544	6.183
		3	5.0135	4.8962	0.315	−5.033	15.060
	2	1	4.6806	5.2944	0.384	−6.183	15.544
		3	9.6940	4.7248	0.050	0.000	19.388

**Table 5:** Pearson correlation analysis between cephalometric variables and OSA parameters (AHI, AHI supine) in the normal-weight group (BMI < 25).

		AHI	AHI Supine
AHI	Pearson correlation	1	0.489
	Sig. (2-tailed)		0.127
	N	15	11
AHI supine	Pearson correlation	0.489	1
	Sig. (2-tailed)	0.127	
	N	11	11
SNA angle	Pearson correlation	−0.292	−0.163
	Sig. (2-tailed)	0.047	0.633
	N	15	11
SNB angle	Pearson correlation	−0.078	0.063
	Sig. (2-tailed)	0.781	0.854
	N	15	11
ANB angle	Pearson correlation	−0.148	−0.207
	Sig. (2-tailed)	0.599	0.542
	N	15	11
FMA angle	Pearson correlation	0.299	0.277
	Sig. (2-tailed)	0.047	0.409
	N	15	11
AO-BO	Pearson correlation	0.159	−0.034
	Sig. (2-tailed)	0.571	0.922
	N	15	11
Occlusal plane angle	Pearson correlation	−0.070	−0.275
	Sig. (2-tailed)	0.803	0.414
	N	15	11
AFH	Pearson correlation	−0.109	0.271
	Sig. (2-tailed)	0.700	0.421
	N	15	11
PFH	Pearson correlation	−0.355	−0.181
	Sig. (2-tailed)	0.050	0.595
	N	15	11
SPAS	Pearson correlation	−0.020	0.294
	Sig. (2-tailed)	0.943	0.381
	N	15	11
PAS	Pearson correlation	0.137	0.173
	Sig. (2-tailed)	0.627	0.610
	N	15	11
MP-H	Pearson correlation	0.201	0.501
	Sig. (2-tailed)	0.050	0.117
	N	15	11
Pns-P	Pearson correlation	0.144	−0.172
	Sig. (2-tailed)	0.609	0.612
	N	15	11
H-C3	Pearson correlation	0.304	0.200
	Sig. (2-tailed)	0.270	0.556
	N	15	11

**Table 6:** Pearson correlation analysis between cephalometric variables and OSA parameters (AHI, AHI supine) in overweight group (BMI > 25).

		AHI	AHI Supine
AHI	Pearson correlation	1	0.489
	Sig. (2-tailed)		0.127
	N	15	11
AHI supine	Pearson correlation	0.489	1
	Sig. (2-tailed)	0.127	
	N	11	11
SNA angle	Pearson correlation	−0.292	−0.163
	Sig. (2-tailed)	0.291	0.633
	N	15	11
SNB angle	Pearson correlation	−0.078	0.063
	Sig. (2-tailed)	0.781	0.854
	N	15	11
ANB angle	Pearson correlation	−0.148	−0.207
	Sig. (2-tailed)	0.599	0.542
	N	15	11
FMA angle	Pearson correlation	0.247	0.277
	Sig. (2-tailed)	0.046	0.409
	N	15	11
AO-BO	Pearson correlation	−0.300	−0.034
	Sig. (2-tailed)	0.046	0.922
	N	15	11
Occlusal plane angle	Pearson correlation	−0.070	−0.275
	Sig. (2-tailed)	0.803	0.414
	N	15	11
AFH	Pearson correlation	0.321	0.271
	Sig. (2-tailed)	0.050	0.421
	N	15	11
PFH	Pearson correlation	−0.375	−0.181
	Sig. (2-tailed)	0.168	0.595
	N	15	11
SPAS	Pearson correlation	−0.280	0.294
	Sig. (2-tailed)	0.050	0.381
	N	15	11
PAS	Pearson correlation	−0.244	0.173
	Sig. (2-tailed)	0.044	0.610
	N	15	11
MP-H	Pearson correlation	0.183	0.501
	Sig. (2-tailed)	0.514	0.117
	N	15	11
Pns-P	Pearson correlation	0.144	−0.172
	Sig. (2-tailed)	0.609	0.612
	N	15	11
H-C3	Pearson correlation	0.304	0.200
	Sig. (2-tailed)	0.270	0.556
	N	15	11

## Data Availability

All data are available upon request to the corresponding author.
